# Optimization of carboxymethylcellulase production from *Bacillus amyloliquefaciens* SS35

**DOI:** 10.1007/s13205-013-0169-6

**Published:** 2013-09-06

**Authors:** Shuchi Singh, Vijayanand S. Moholkar, Arun Goyal

**Affiliations:** 1 Center for Energy, Indian Institute of Technology Guwahati, Guwahati, 781 039 Assam India; 2 Department of Chemical Engineering, Indian Institute of Technology Guwahati, Guwahati, 781 039 Assam India; 3 Department of Biotechnology, Indian Institute of Technology Guwahati, Guwahati, 781 039 Assam India

**Keywords:** Carboxymethylcellulase, *Bacillus amyloliquefaciens*, Medium optimization, Fermentation parameter optimization, Central composite design

## Abstract

In this paper, we have attempted optimization of production of enzyme carboxymethylcellulase or endoglucanase from the bacterium *Bacillus amyloliquefaciens* SS35. Optimization has been carried out in two stages using statistical experimental design, viz. medium optimization and optimization of fermentation parameters. For medium optimization, Plackett–Burman design followed by central composite design (CCD) was used, while for optimization of fermentation parameters one-variable-at-a-time method followed by CCD was used. Carbon and nitrogen sources in the medium have been revealed to be the significant factors for enzyme production (carboxymethylcellulose 18.05 g/L; yeast extract 8 g/L and peptone 2 g/L). The inorganic salts have been found to be insignificant components of medium. Optimum fermentation parameters for optimized medium were: initial medium pH 5.65, incubation temperature = 40 °C, shaking speed = 120 rpm, and inoculum size = 6.96 %, v/v. Interestingly, the influence of all four parameters was almost independent with no interlinks. Secondly, the overall effect of all parameters was also low, as indicated by linear, square and interaction regression coefficients that were at least one order of magnitude lower than the intercept in the model equation. These results essentially meant that medium components dominate overall enzyme production process in comparison to fermentation parameters.

**Electronic supplementary material:**

The online version of this article (doi:10.1007/s13205-013-0169-6) contains supplementary material, which is available to authorized users.

## Introduction

Use of cellulases in bioethanol production from various lignocellulosic substrates has been reported by many authors recently (Ballesteros et al. 2004; Kuhad et al. 2010; Mutreja et al. 2011; Rodhe et al. 2011). Cellulases essentially convert cellulose fraction of lignocellulosic biomass to fermentable sugars. Cellulases, which essentially are the suite of enzymes that act synergistically for complete hydrolysis of cellulose to glucose, include: endoglucanases (carboxymethylcellulase, E.C. 3.2.1.4), exoglucanases (avicelase, E.C. 3.2.1.91), and cellobiases (β-glucosidase, E.C. 3.2.1.21) (Lynd et al. 2002). Among the group of cellulase enzymes, carboxymethylcellulase (CMCase) has been used predominantly in the process of ethanol production (Ballesteros et al. 2004; Zheng et al. 2009; Singh and Bishnoi 2012). Commercial cellulases are mainly produced by fungi (e.g., *Aspergillus* and *Trichoderma* sp.); however, bacteria have also been considered as robust and versatile enzyme producers because of their high growth rate, stability at extreme conditions, and presence of multi-enzyme complexes. Among these, *Bacillus* sp. has been revealed to be the most potent extracellular enzyme producer (Singh et al. 2001; Pason et al. 2006; Khan and Husaini 2006; Lee et al. 2008; Van Dyk et al. 2009; Kim et al. 2009). Enzymatic hydrolysis of biomass is an important step in the production of bioethanol. Enzyme being an expensive component of this process, the economical production of cellulase is an important aspect of the overall economic feasibility of bioethanol production. The extent of enzyme production by a bacterial culture is a function of fermentation parameters and components of the medium. Optimization of these parameters is a key factor influencing the economic feasibility of enzymatic hydrolysis of biomass prior to fermentation. In this study, the medium of *Bacillus amyloliquefaciens* SS35 culture was optimized for CMCase production using statistical design of experiments. This cellulolytic bacterium has been isolated by us from rhinoceros dung. The conventional optimization technique for the medium as well as fermentation parameters is one-variable-at-a-time (OVAT) method. However, this method has limitations in which it is not able to identify the combined or interactive effect of different components of the medium or different parameters of fermentation process. Statistical experimental designs such as central composite experimental design fitted with second-order model can be applied to overcome this limitation. In this study, we have addressed the matter of optimization of medium as well as fermentation parameters (including fermentation parameters like pH, temperature, inoculum size, and shaking speed) for the *B. amyloliquefaciens* SS35 to have enhanced enzyme production. For the optimization of medium composition, considering large number of possible medium components, we have employed a two-stage methodology of initial screening of all components using Plackett–Burman design. This design identifies the components having most significant influence on response variable, i.e., CMCase production. This is followed by a central composite design (CCD) for identification of optimum values of the significant components identified earlier for enhanced production of enzymes. The regression coefficients and ANOVA of the second-order model also help in identifying the interaction between significant medium components. For optimization fermentation parameters, we have used the CCD design preceded by OVAT method.

## Materials and methods

### Materials

Carboxymethylcellulose (CMC) (low viscosity, 50–200 cP) was procured from Sigma-Aldrich (St. Louis, USA), and medium components (listed in next section) were procured from HiMedia Pvt. Ltd., India.

### Microorganism and culture conditions

The bacterium used in this study, *B. amyloliquefaciens* SS35 (GenBank accession no.: JX674030), was isolated from rhinoceros dung (Singh et al. 2013), and the bacterial culture was maintained on nutrient agar slants. The basic (unoptimized) medium composition, used for CMCase production under unoptimized conditions, were CMC-Na (10 g/L), yeast extract (5 g/L), peptone (5 g/L), K_2_HPO_4_ (1 g/L), MgSO_4_·7H_2_O (0.2 g/L) and NaCl (1 g/L). The unoptimized fermentation conditions were incubation temperature 37 °C, initial medium pH 7.0, shaking speed 180 rpm, and inoculum size 2 %, v/v (Optical density at 600 nm = 1.0).

### Experiments for medium optimization

The culture was grown in enzyme production medium (pH 7.0) containing following six components: CMC-Na, yeast extract, peptone, K_2_HPO_4_, MgSO_4_·7H_2_O, and NaCl. These medium components were selected on the basis on published literature on CMCase production from different *Bacillus* sp. (Apun et al. 2000; Singh et al. 2001; Lee et al. 2008; Kim et al. 2009; Rastogi et al. 2009; Deka et al. 2011). Two levels of concentrations of each of these components (again based on the published literature) were selected for Plackett–Burman analysis. The total number of experiments, with permutation–combination of different components and their levels, were 20. The details of these experiments are given in Table S1 in the supplementary material provided with this paper. In each of these 20 experiments, three trial runs have been taken so as to assess the reproducibility of results. In each experiment, 100-mL medium containing 2 % (OD_600nm_ = 1.0) inoculum was taken in a 250-mL Erlenmeyer flask and incubated at 37 °C with shaking at 180 rpm for 72 h in a shaking incubator (Model: Orbitek, Make: Scigenics Biotech). After every 6 h, the culture broth was centrifuged at 12,000*g* for 20 min at 4 °C. The cell-free supernatant containing the crude enzyme was used for estimation of CMCase activity (the enzyme assay has been explained subsequently). It was observed that the CMCase activity showed maxima after 48 h of incubation, and a reduction in activity was seen thereafter. In view of this, the activity at 48 h was considered for analysis. The cell growth was monitored by taking OD at 600 nm using UV–visible spectrophotometer (Varian, Model Cary-100).

### Experiments for fermentation parameters optimization

The optimization parameters in this category were: (1) incubation temperature (°C), (2) initial pH of the medium, (3) shaking speed (rpm), and (4) inoculum size (%, v/v). The optimized medium (defined subsequently) was used for further optimization of fermentation parameters, and the initial pH of the medium was adjusted to the selected range (pH 4.5–11.0) in different sets of experiments using 2 N HCl and NaOH. In this case, we have adopted the CCD experimental design preceded by OVAT analysis to determine the levels of these parameters. The CCD experimental design and its analysis are based on these levels. The exact experimental design employed is described in Table S2 given as supplementary material.

### CMCase assay

The response variable of the statistical experiments is CMCase activity (U/mL), which was quantified on the basis of measurement of total reducing sugar liberated after incubation of enzyme with CMC. The enzyme assay was carried out by incubating the enzyme (cell-free supernatant) with CMC for 15 min at 55 °C. The reaction mixture (100 μL) contained 20 μL of enzyme and 1 % (w/v) final concentration of CMC in 50-mM sodium acetate buffer (pH 5.0). The total reducing sugar was quantified using method of Nelson and Somogyi (Nelson 1944; Somogyi 1945). The absorbance was measured at 500 nm using a UV–visible spectrophotometer (Varian, Model Cary-100) against a blank with d-glucose as standard. One unit (U) of cellulase activity is defined as the amount of enzyme that liberates 1 μmol of reducing sugar (glucose) in 1 min at 55 °C and pH 5.0.

### Experimental designs for medium optimization

#### Plackett–Burman design

As stated earlier, the Plackett–Burman experimental design was used for initial screening of medium components to short-list components having significant effect on CMCase production. This experimental design was devised using statistical software package MINITAB (Release 15.1, PA, USA, Trial Version). Plackett–Burman factorial design is a two-level fractional design that follows first-order polynomial model:1Notations: *Y* response variable (CMCase activity), *β*_o_ model intercept, *β*_*i*_ linear coefficient, and *X*_*i*_ level of independent variable. Totally six medium components (or factors) have been examined at two levels each, viz. −1 and +1, indicating low and high level, respectively (Plackett and Burman 1946). These components (or factors) and their coded as well as actual values (given in parentheses) are shown in Table S1. This experimental design comprised of a total of 20 sets of experiments (with each experiment conducted in triplicate to assess reproducibility). The average CMCase activity in each experiment was considered as the response. The regression analysis of Plackett–Burman design revealed the significant variables (or medium components) influencing CMCase activity, with significance level ≥95 % and *p* value <0.05 (Table 1a). This result was further corroborated with CCD of experiments.

**Table 1:** Statistical analysis for the results from Plackett–Burman experimental design

Model term	Coefficient estimate	Computed *t* value	*p* value	Confidence level (%)	
(a) Coefficient values, *t* and *p* value for each variable					
Intercept	0.33265	51.00	0*	100	
CMC (*X*_1_)	0.11625	17.82	0*	100	
Yeast extract (*X*_2_)	0.04215	6.46	0*	100	
Peptone (*X*_3_)	0.02675	4.10	0.001*	99.9	
K_2_HPO_4_ (*X*_4_)	0.00755	1.16	0.268	73.2	
MgSO_4_·7H_2_O (*X*_5_)	−0.01105	−1.69	0.114	88.6	
NaCl (*X*_6_)	0.00755	1.16	0.268	73.2	

Variable	SS	DF	MS	*F* value	*p* value
					prob > *F*
(b) ANOVA for the model					
Constant	0.325	6	0.0541	63.62	0
CMC (*X*_1_)	0.27	1	0.27	317.59	0
Yeast extract (*X*_2_)	0.0355	1	0.0355	41.75	0
Peptone (*X*_3_)	0.0143	1	0.0143	16.82	0.001
K_2_HPO_4_ (*X*_4_)	0.00114	1	0.00114	1.34	0.268
MgSO_4_·7H_2_O (*X*_5_)	0.00244	1	0.00244	2.87	0.114
NaCl (*X*_6_)	0.00114	1	0.00114	1.16	0.268
Residual	0.0111	13	0.000851	1.34	
Total	0.336	19			

*DF* degrees of freedom, *SS* sum of squares, *MS* mean square

* Significant *p* values, *p* ≤ 0.05; *R*^2^ = 0.9671; predicted *R*^2^ = 0.9220; adjusted *R*^2^ = 0.9519

#### Central composite design

The central composite experimental design is commonly employed for processes with significant interaction effects between variables. This design essentially fits a second-order polynomial model. We have used a 3-factor-5-level design, in which five coded levels (−*α*, −1, 0, +1, +*α*) were assigned to each factor. α is the extended level with value of (2)^3/4^ = 1.682. A 2^3^ full-factorial CCD experimental design with three significant medium constituents (viz., CMC, yeast extract and peptone, as explained subsequently) at five coded levels was generated by Minitab statistical software (Release 15, Trial Version). This experimental design comprised of 20 individual experiments (=2^*k*^ + 2*k* + *n*_o_), where ‘*k*’ is the number of independent variables and *n*_o_ is the number of replicate runs at center point of the variables. This design is described in Table S2.

#### Statistical analysis and model fitting

The experimental data (Table S2) were analyzed by the regression procedure to fit the following second-order polynomial equation:2 Notation: *Y* response (CMCase activity), *k* number of factors or medium components, *β*_o_ regression constant, *β*_*i*_ linear coefficient, *β*_*ii*_ quadratic coefficient, and *β*_*ij*_ interaction coefficient. The following equation was used for coding (in the range of −1 to +1) the actual experimental values (given in the parentheses in the table) of the factors:3where, *x*_*i*_ is the dimensionless value of an independent variable, *X*_*i*_ is the real value of an independent variable, *X*_o_ is the value of *X*_*i*_ at the center point, and Δ*X*_*i*_ is the step change. The analysis of variance (ANOVA) was also done to determine the significance of each factor in fitted model Eq. (2), and also to determine the goodness of fit. For this purpose, the software Design Expert 7.0 (trial version) has been used. Graphical representation of the fitted polynomial Eq. (2) has been given in the form of contour plots. ANOVA of the linear, quadratic and interaction regression coefficients (i.e., *F* value and *p* value) has been given in Table 2. The *F* and *p* values essentially exhibit the individual and interactive effects of the independent variables.

**Table 2:** Results of statistical (CCD) analysis for medium optimization

Model term	Coefficient estimate	Computed *t* value	*p* value	Confidence level (%)	
			prob > *F*		
(a) Model coefficient estimated by regressions					
Intercept	0.443596	56.21	0*	100	
CMC (*X*_1_)	0.087215	16.657	0*	100	
Yeast extract (*X*_2_)	−0.028983	−5.535	0*	100	
Peptone (*X*_3_)	−0.008664	−1.655	0.129	87.1	
CMC × CMC	−0.045706	−8.967	0*	100	
Yeast extract × yeast extract	0.008741	1.715	0.117	88.3	
Peptone × peptone	−0.011942	−2.343	0.041*	95.9	
CMC × yeast extract (*X*_1_ × *X*_2_)	0.0175	2.558	0.028*	97.2	
CMC × peptone (*X*_1_ × *X*_3_)	0.02175	3.179	0.01*	99	
Yeast extract × peptone (*X*_2_ × *X*_3_)	0.02975	4.349	0.001*	99.9	

Source	DF	SS	MS	*F* value	*p* value
					prob > *F*
(b) ANOVA for quadratic model					
Regression	9	0.163	0.0182	48.51	0
Linear	3	0.116	0.0388	103.61	0
Square	3	0.0338	0.0113	30.05	0
Interaction	3	0.0133	0.00444	11.85	0.001
Residual (error)	10	0.00374	0.000374		
Lack of fit	5	0.00281	0.000561	2.99	0.127
Pure error	5	0.000938	0.000188		
Total	19	0.167			

*DF* degrees of freedom, *SS* sum of squares, *MS* mean square

* Significant *p* values, *p* ≤ 0.05; *R*^2^ = 0.9776; predicted *R*^2^ = 0.8642; adjusted *R*^2^ = 0.9575

### Experimental design for optimization of fermentation parameters

#### Central composite design

A 2^4^ full-factorial CCD experimental design with four parameters (viz., incubation temperature, initial medium pH, rpm or speed of orbital shaking and inoculum size) at five coded levels was generated by Minitab statistical software (Release 15, Trial Version) (Table S3 provided in supplementary material). In this 4-factor-5-level design, five coded levels (−*α*, −1, 0, +1, +*α*) were assigned to each factor, where *α* = (2)^4/4^ = 2. This experimental design comprised of 31 individual experiments (=2^*k*^ + 2*k* + *n*_o_), where *k* is the number of independent variables (4) and *n*_o_ being the number of replicate runs (7) at center point of the variables.

### Experimental validation of optimization

Experiments have been conducted in two steps with optimized medium composition and optimized fermentation parameters predicted by statistical analysis to assess the accuracy of the models. Initially, validation of optimization of medium composition was carried out using results of CCD experimental design. Next, validation of optimum fermentation parameters (at optimum medium composition) has been carried out. To assess the reproducibility, all validation experiments have been performed in triplicate.

## Results and discussion

As noted earlier, we have carried out optimization of CMCase production by the bacterium *B. amyloliquefaciens* SS35 in two stages, viz. optimization of the medium, and secondly, optimization of the fermentation parameters. In accordance with this, we first present the results of medium optimization followed by results of optimization of fermentation parameters. Finally, we try to link the results of these two studies.

### Optimization of fermentation medium

#### Plackett–Burman design for screening of significant medium components

The results of initial screening of medium components using Plackett–Burman design are given in Table S1. The experimental and predicted values of CMCase activity match quite well. The CMCase activity in the cell-free supernatant of *B. amyloliquefaciens* SS35 varied from 0.132 to 0.528 U/mL. As stated in previous section, this is the maximum activity attained after 48 h of incubation. The statistical analysis of the Plackett–Burman design is given in Table 1a, b. The values of the first-order model coefficients for all six variables along with *t* value, *p* value, and confidence levels are depicted in Table 1a, while the ANOVA of the model is given in Table 1b. The model variable is termed to be significant if the *t* value is greater than *p* value. As seen from Table 1a, this condition is satisfied by three variables, viz CMC, yeast extract, and peptone. The significance of these variables is also corroborated by results of Table 1b, which shows high *F* value and zero *p* value for these variables. The *t* value limit for this analysis is 2.16 (as shown in Pareto chart Figure S1, given in supplementary material). The *t* value for CMC, yeast extract, and peptone are higher than this limit, which points to their significance. Moreover, MgSO_4_·7H_2_O has shown a negative effect on the CMCase activity as indicated by the negative Plackett–Burman model coefficient. Another measure of significance level of variables is the confidence level, which is ~100 % for CMC, yeast extract, and peptone. The overall regression coefficient for the Plackett–Burman design is *R*^2^ = 0.9671, with adjusted *R*^2^ = 0.9519 shows the model fits very well to the data. Neglecting the insignificant variables, the model equation for CMCase activity is written as:4 Among the significant variables CMC acts as carbon source, while yeast extract and peptone are the nitrogen sources. Earlier studies by Li et al. (2008) and Deka et al. (2011) report significant role played by these components on cellulase production by *Bacillus* sp. The inorganic salts have been found to have insignificant effect on CMCase production. This is rather an anomaly as potassium phosphate is known for the production of microbial polysaccharides and also as an ingredient in buffer solutions that enhance cell growth (Lee et al. 2008; Jin et al. 2011; Gao et al. 2012); sodium chloride as well as magnesium sulphate in the medium play important role in initial cell growth. We explain this anomaly in terms of differences in the phase of cell life cycle in which cell growth and enzyme production occurs. The maximum CMCase production was observed at late log phase or early stationary phase of life cycle (Singh et al. 2013).

#### Optimization by central composite experimental design

The CCD was based on the results of Plackett–Burman design, which revealed that three factors, viz. CMC, yeast extract, and peptone had significant influence on CMCase production. The full factorial CCD matrix of these variables is given in Table S2 supplementary material provided. Table S2 also gives the CMCase activity obtained in each experiment and the standard deviation. The results of second-order response model fitted to the coded data are given in Table 2a. The second-order regression equation fitted to this data is as follows:5Notations are same as that for Plackett–Burman design. The overall model had regression coefficient of 0.9776 with adjusted coefficient of *R*^2^ = 0.9575. The predicted *R*^2^ of 0.8642 is also in reasonable agreement with adjusted *R*^2^ of 0.9575. This essentially indicates that the model fits very well to the experimental data. This is also corroborated by the predicted CMCase activity values (listed in Table S2 of supplementary material) that match well with the experimental values. The ANOVA of the fitted model is described in Table 2b. The lack of fit *F* value of 2.99 implies that lack of fit is not significantly relative to pure error. The *p* value of 0.127 for lack of fit implies that there is 12.7 % chance that a ‘lack of fit *F* value’ this large could occur due to noise. Another yardstick for fitness of model is Adeq Precision value that measures the signal-to-noise ratio. A ratio >4 is desirable. The ratio obtained for the present analysis is 25.538, which represents adequacy of the signal. The *p* value of the linear, square and interaction coefficients are all zero pointing their significance. An interesting trend is in the *F* values of the coefficients. The *F* value for linear coefficients is far higher (>10×) than the value for interaction coefficients. This essentially means that effects of CMC, yeast extract and peptone on the CMCase production are rather independent, with no interlinks between them.

The optimum value of concentration of medium components as revealed from the statistical analysis and quadratic model for maximum CMCase production are CMC = 19.05 g/L, yeast extract = 8 g/L, and peptone = 2 g/L. These have been depicted in the desirability function plot shown in Figure S2 of supplementary material. The maximum CMCase activity for these parameters the model predicts 0.585 U/mL. The experiments were conducted to validate these predictions and the CMCase activity for the optimum medium composition was found to be 0.553 ± 0.021 U/mL, which is in close agreement with predicted result of CCD. The CMCase activity under unoptimized conditions (as mentioned in section Microorganism and culture conditions) was 0.161 ± 0.014 U/mL; thus, the resulted enhancement in enzyme activity after medium optimization was about 3×.

#### Interaction effects of medium components

**Fig. 1:**
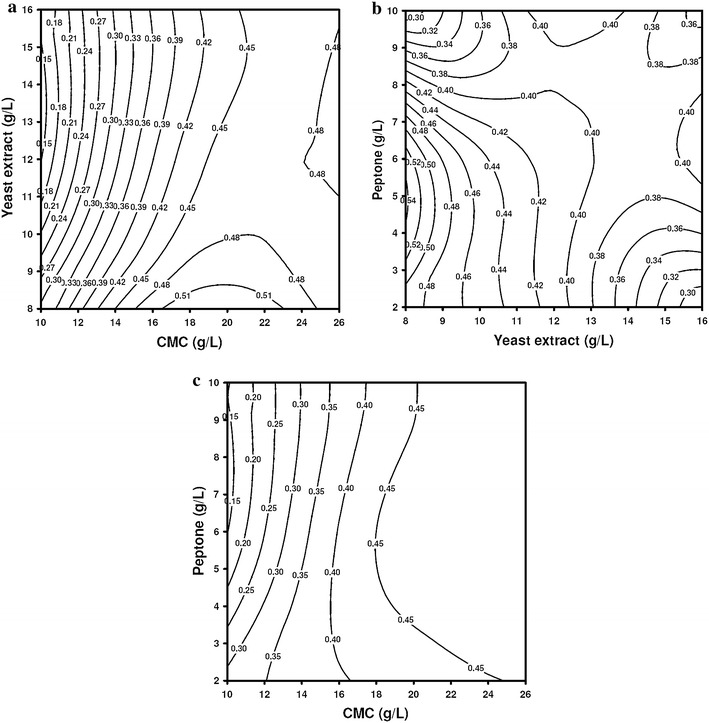
Contour plots for CMCase production showing the interactive effects of medium components: **a** concentrations of yeast extract and CMC, **b** concentrations of yeast extract and peptone, **c** concentrations of CMC and peptone

The interactive effects between medium components have been assessed using contour plots shown in Fig. 1. Following trends in interactive effect can be identified: (1) for smaller concentration of both yeast extract and CMC, the enzyme activity increases with concentration. However as the CMC concentration crosses 16 g/L, the highest activity is seen for low concentration of yeast extract ~8 g/L. (2) The highest activity is seen for the yeast extract concentration of 8 g/L and peptone concentration range of 3–6 g/L. With concentration of peptone and yeast extract increasing beyond this range, the activity reduces. (3) The CMCase activity increases monotonically with both peptone and CMC concentration.

We explain these results on the basis of amino acid composition of the enzyme CMCase (endoglucanase) given in supplementary material. On the basis of the methodology given by Unrean and Nguyen (2013), we have also listed (Table S4 in supplementary material) the metabolic and energy requirement to produce one mole of CMCase by *B. amyloliquefaciens*. Based on the amino acid composition it could be seen that one mole of CMCase production requires large amount of nitrogen, i.e., 677 mol of ammonia. Consequently, both peptone and yeast extract being the nitrogen sources play a vital role in CMCase production. CMC being the main substrate of the metabolic pathway enhances bacterial cell growth, and thus, a significant component of the process.

### Optimization of fermentation parameters

**Fig. 2:**
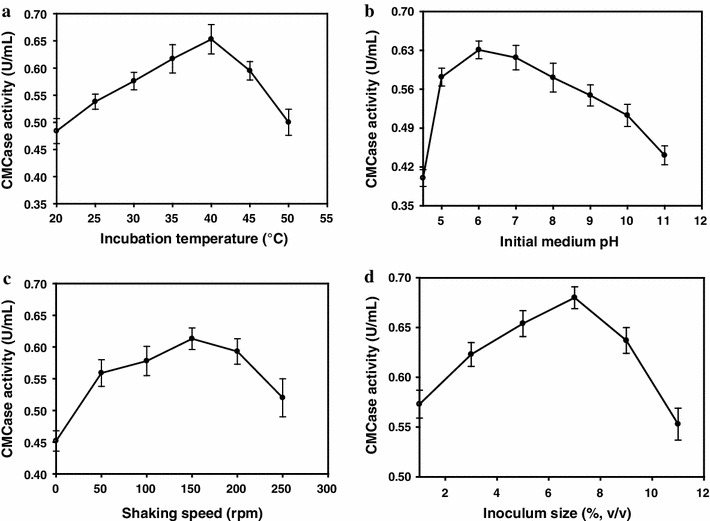
Evaluation of individual effect of fermentation parameters on CMCase production (one-variable-at-a-time method): **a** effect on incubation temperature at medium pH 7.0, shaking speed = 180 rpm, and inoculum size = 2 % v/v; **b** effect of initial medium pH at temperature = 37 °C, shaking speed = 180 rpm, and inoculum size = 2 % v/v; **c** effect of shaking speed at medium pH 7.0, temperature = 37 °C, inoculum size = 2 % v/v; **d** effect of inoculum size at temperature = 37 °C, shaking speed = 180 rpm, and medium pH  7.0

As noted earlier, the optimization of fermentation parameters was carried out in two steps: (1) OVAT method, and (2) central composite experimental design. As reported in literature (for example, Immanuel et al. 2006), the cellulase production is a function of several parameters such as temperature, initial pH of the medium, and inoculum size. The OVAT experiments helped in arrangement of individual effect of the fermentation parameters, as well as in deciding the levels of these parameters for a CCD design. The results of OVAT experiment are depicted in Fig. 2. It could be seen that each of the parameters shows an optimum value at which maxima of CMCase activity is observed. The values of these parameters are (1) temperature = 40 °C, (2) initial pH of medium = 6.0, (3) shaking speed = 150 rpm, and (4) inoculum volume = 6 % v/v.

#### Optimization of fermentation parameters by CCD

The CCD experimental matrix with the actual and coded values of the above parameters is given in Table S3 provided in supplementary material, along with the values of response variable for each experiment with different combination of parameters. The results of second-order response model fitted to the coded data and the ANOVA of the model is given in Table 3a, b. The model equation for response variable is as follows:

**Table 3:** Results of statistical (CCD) analysis for optimization of fermentation parameters

Model term	Coefficient estimate	Computed *t* value	*p* value	Confidence level (%)	
(a) Model coefficient estimated by regressions					
Intercept	0.637714	184.607	0*	100	
Temperature (*X*_1_)	0.008208	4.4	0*	100	
Medium pH (*X*_2_)	−0.021208	−11.368	0*	100	
Shaking (*X*_3_)	−0.006125	−3.283	0.005*	99.5	
Inoculum size (*X*_4_)	0.004958	2.658	0.017*	98.3	
Temperature × temperature	−0.028564	−16.713	0*	100	
pH × pH	−0.007314	−4.279	0.001*	99.9	
Shaking × shaking	0.008811	5.155	0*	100	
Inoculum size × inoculum size	−0.002689	−1.573	0.135	86.5	
Temperature × medium pH (*X*_1_ × *X*_2_)	0.003563	1.559	0.139	86.1	
Temperature × shaking (*X*_1_ × *X*_3_)	−0.007438	−3.255	0.005*	99.5	
Temperature × inoculum size (*X*_1_ × *X*_4_)	0.005938	2.599	0.019*	98.1	
Medium pH × shaking (*X*_2_ × *X*_3_)	0.001187	0.520	0.610	39	
Medium × inoculum size pH (*X*_2_ × *X*_4_)	0.000313	0.137	0.893	10.7	
Shaking × inoculum size (*X*_3_ × *X*_4_)	0.000063	0.027	0.979	2.1	

Source	DF	SS	MS	*F* value	*p* value
					prob > *F*
(b) ANOVA for quadratic model					
Regression	14	0.043830	0.003131	37.48	0
Linear	4	0.013903	0.003476	41.61	0
Square	4	0.028251	0.007063	84.55	0
Interaction	6	0.001676	0.000279	3.34	0.025
Residual (error)	16	0.001337	0.000084		
Lack of fit	10	0.001133	0.000113	3.34	0.077
Pure error	6	0.000203	0.000034		
Total	30	0.045167			

*DF* degrees of freedom, *SS* sum of squares, *MS* mean square

* Significant *p* values, *p* ≤ 0.05; *R*^2^ = 0.9701; predicted *R*^2^ = 0.9422; adjusted *R*^2^ = 0.8473

6 It could be seen that all coefficients (linear/quadratic/interaction) are at least one to three orders of magnitude smaller than the intercept value. The analysis of coefficients presented in Table 3a shows that most of linear and quadratic coefficients are significant (as indicated by *p* < 0.05), while most of interaction coefficients are insignificant. The overall model fits very well to the data as seen from *R*^2^ value of 0.9704 and fairly matching values of *R*^2^ = 0.8494. ANOVA of the model presented in Table 3b shows all regression coefficients as significant. But relative magnitude of *F* values of these coefficients indicates that individual influence of parameters is much higher than interactive effect.

#### Interaction effects of fermentation parameters

**Fig. 3:**
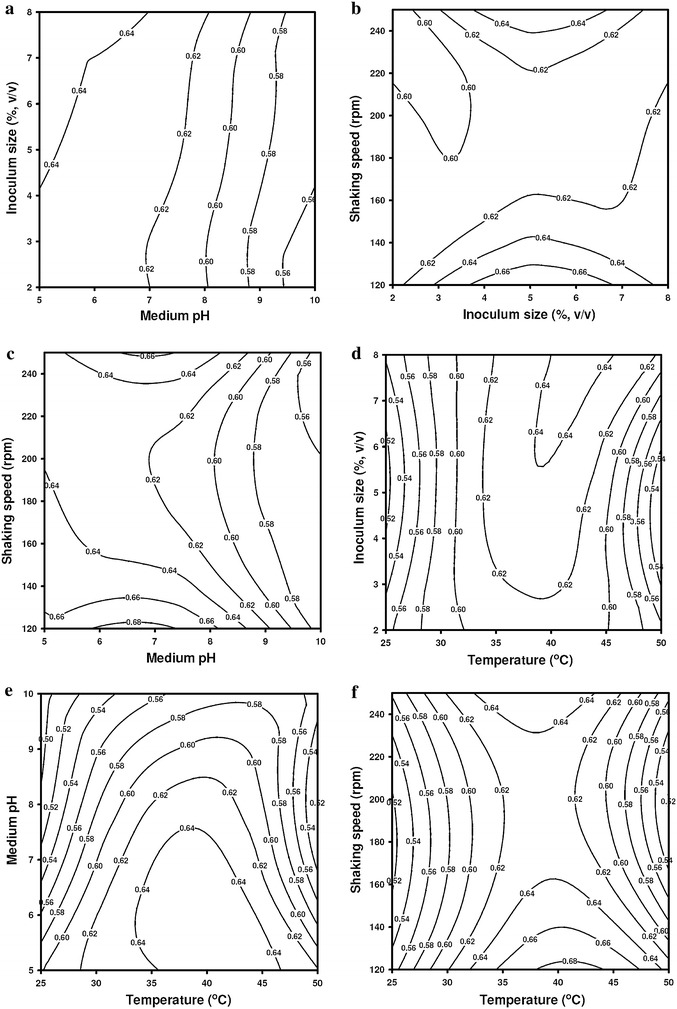
Contour plot for CMCase production showing the interactive effects of fermentation parameters: **a** medium pH and inoculum size; **b** shaking speed and inoculum size; **c** shaking speed and medium pH; **d** temperature and inoculum size; **e** temperature and medium pH; **f** temperature and shaking speed

The interaction between the fermentation parameters has been explained with the help of contour plots of the regression model, shown in Fig. 3a–f. The highest CMCase activity is seen for temperature range of 35–45 °C with corresponding pH range of 5.0–7.0. At a given temperature, even twofold variation of shaking speed does not give a significant rise in the activity. Similarly, in the optimum pH range of 6.0–7.0, shaking speed does not influence CMCase activity. Same observation holds true for variation of CMCase activity with inoculum size and pH. For the combination of pH and temperature, CMCase activity is in the temperature range of 40–45 °C and medium pH of 6.0–7.0. The activity rapidly reduces as either of this variable shift away from the optimum range. Nonetheless, an overall observation seen in all contour plots is that with twofold, threefold or even fourfold variation of each fermentation parameter; the activity shows a marginal variation of ±20–40 %. The optimum values of the four variables that could be deduced from the desirability function plot shown in Figure S3 (in supplementary material) are temperature = 40 °C, pH 5.65, shaking speed = 120 rpm, and inoculum size = 6.9 % v/v. The predicted enzyme activity at these conditions was 0.710 U/mL. Validation of these results was done using shake flask experiments at the optimum values of different parameters. The experimental enzyme activity was observed to be 0.693 ± 0.043 U/mL, which is an excellent agreement with the predicted value. Quite interestingly these values match fairly well with the values obtained from OVAT analysis. The observed enhancement of CMCase activity (0.693 U/mL) after complete optimization (i.e., medium composition and fermentation parameters) was about fourfold in comparison to activity (0.161 U/mL) under unoptimized conditions. However, the increment in activity after optimization of medium composition alone was threefold, and further optimization of fermentation parameters yielded only onefold increment. Thus, it can be concluded that the concentration of major medium components is the principal governing factor for CMCase production. The outcome of the study by Deka et al. (2013) also supports this conclusion, where total optimization of medium composition and fermentation parameters resulted to eightfold enhancement in CMCase activity, out of which the contribution of medium optimization alone to enhancement was sixfold. Optimization of CMCase production by *Bacillus* sp. has been studied by earlier authors using strains isolated from different sources. A comparative account of this is given in Table 4a, b for optimization of medium as well as optimization of fermentation parameters. The activity reported by Lee et al. (2008, 2010) for their *Bacillus* sp. is significantly higher (i.e., 137 and 153 U/mL) than that reported in this study as well as several other studies cited in Table 4a. However, it should noted that the Lee et al. (2008, 2010) have determined the activity on bioreactor scale (as compared to shake flask scale in present study), which a far better and precisely controlled system in terms of operating parameters. Moreover, the sole carbon source for all species reported in Table 4a is different from each other, which has influence on the actual activity of the enzyme. For example, Sethi et al. (2013) have used glucose as the sole carbon source, while Shabeb et al. (2010) have used dual carbon source in the form of molasses and cellulose. It should be pointed out that bacteria producing cellulase with activity in a similar range as for our species could hydrolyze several lignocellulosic biomass significantly, depending the substrate specificity of enzyme. Examples are: (1) Wild grass (*Achnatherum hymenoides*) hydrolysis by *Bacillus subtilis* AS3 with CMCase activity 0.57 U/mL (Deka et al. 2013); (2) Sago pith waste hydrolysis by *B. amyloliquefaciens* UMAS 1002 with CMCase activity 0.63 U/mL (Apun et al. 2000); (3) enzymatic hydrolysis of cellulosic biomass like sugarcane bagasse for bioethanol production using cellulase with lower activity (i.e., 0.112 and 0.902 U/mL) produced by *T. longibrachiatum* PTCC 5140 and *Aspergillus niger*, respectively (Shaibani et al. 2011). We would also like to point out that Kabel et al. (2006) have proven with rigorous study of activity of numerous commercial enzyme preparations (from suppliers such as Novozymes, Dyadic, Genencor, Rhodia-Danisco, Lyven) comprising of cellulase, cellobiase, and xylanases that the activities predicted by standard assays do not really reflect the actual activity of the enzyme on all substrates. The actual activity is more substrate dependent—in that enzymes showing lower activity with standard assays can do efficient hydrolysis with a particular substrate. For convenience of the reader, we briefly reproduce here the overall outcome of the study of Kabel et al. (2006). Activity analyzed in the standard xylanase test did not present a high correlation with the degradation of xylans in the xylan-rich KOHss fractions of wheat bran and grass. A remarkable example is the comparison of the standard activity of Cellubrix (107 U/mL) and Cellulase 2000L (568 U/mL) with the degradation of xylan to xylose of Cellubrix (only 50–60 %) and Cellulase 2000L (only 18–20 %). The enzyme activity towards the cellulose- and xylan-enriched fractions from grass and wheat bran was revealed to be markedly different than that found with standard assays. Therefore, the actual degradation of the xylan- and cellulose-rich fractions from wheat bran and grass could not be correlated with the (relatively low) activity of enzymes as indicated by standard assays. Therefore, in general, the choice of most suitable enzyme preparation is dependent on the substrate characteristics rather than on standard enzyme activities measured.

**Table 4:** Comparison of various optima reported in literature for CMCase production by *Bacillus* sp.

*Bacillus* sp.	Source of isolation	Medium component to optimize	Method	Optimum conc. (g/L)	Scale of experiments	Cellulase activity (U/mL)	References
(a) Representative literature review for optimization of medium components							
*B. subtilis*	Soil	Glucose	OVAT	50	Shake flask	1.0	Sethi et al. (2013)
		Ammonium sulphate		5			
		–		–			
*B. subtilis* AS3	Cow dung	CMC	Plackett–Burman and CCD	18	Shake flask	0.43	Deka et al. (2011)
		Yeast extract		4.79			
		Peptone		8			
*B.* sp. VG1	Soil from hot spring	CMC	OVAT	10	Shake flask	0.63	Singh et al. (2001)
		Tryptone		5			
		–		–			
*B. subtilis* subsp. *subtilis* A-53	Sea water	Rice bran	OVAT	20	7-L Bioreactor	137	Lee et al. (2010)
		Yeast extract		2.5			
		–		–			
*B. amyloliquefaciens* DL3	Soil	Rice hull	OVAT	20	7-L Bioreactor	153	Lee et al. (2008)
		Peptone		2.5			
		Ammonium sulphate		0.6			
*B. Subtilis* KO	Product of sugar factory	Molasses + cellulose	OVAT	1.0	Shake flask	35	Shabeb et al. (2010)
		(NH_4_)_2_ PO_4_ or tryptone		2.0			
		–		–			
*B. pumilus* EB3	Oil palm empty fruit bunch	CMC	OVAT	10	Shake flask	0.076	Ariffin et al. (2008)
		Yeast extract		2.5			
		Ammonium sulphate		2.5			
*B. amyloliquefaciens* SS35	Rhinoceros dung	CMC	Plackett–Burman and CCD	19.05	Shake flask	0.55	This study
		Yeast extract		8.0			
		Peptone		2.0			

*Bacillus* sp.	Source of isolation	Optimization parameter	Method	Optimum value	Scale of experiments	Cellulase activity (U/mL)	References
(b) Representative literature review for optimization of fermentation parameters							
*B. amyloliquefaciens* UMAS1002	Sago pith waste	pH	OVAT	6.0	Shake flask	9.38	Khan and Husaini (2006)
		Temperature (°C)		40			
		Inoculum size (%, v/v)		4			
		Shaking speed (rpm)		100			
*Bacillus* sp.	Coir retting effluents	pH	OVAT	7.0	Shake flask	0.0196	Immanuel et al. (2006)
		Temperature (°C)		40			
		Inoculum size (%, v/v)		ND			
		Shaking speed (rpm)		ND			
*B. amyloliquefaciens* UNPDV-22	Hot spring	pH	CCD	5.25	Shake flask	13	Vasudeo and Lew (2011)
		Temperature (°C)		42.24			
		Inoculum size (%, v/v)		4.95			
		Shaking speed (rpm)		ND			
*B. subtilis* AS3	Cow dung	pH	CCD	7.2	Shake flask and 2-L bioreactor	0.56 (shake flask) and 0.75 (bioreactor)	Deka et al. (2013)
		Temperature (°C)		39			
		Inoculum size (%, v/v)		121			
		Shaking speed (rpm)		ND			
*B. subtilis*	Soil	pH	OVAT	10.0	Shake flask	0.9	Sethi et al. (2013)
		Temperature (°C)		40			
		Inoculum size (%, v/v)		ND			
		Shaking speed (rpm)		ND			
*B. subtilis* subsp. *subtilis* A-53	Sea water	pH	OVAT	6.8	7-L Bioreactor	137	Lee et al. (2010)
		Temperature (°C)		30			
		Inoculum size (%, v/v)		ND			
		Shaking speed (rpm)		ND			
*B. amyloliquefaciens* DL3	Soil	pH	OVAT	6.8	7-L Bioreactor	367	Lee et al. (2008)
		Temperature (°C)		37			
		Inoculum size (%, v/v)		ND			
		Shaking speed (rpm)		ND			
*B. amyloliquefaciens* SS35	Rhinoceros dung	pH	CCD	5.65	Shake flask	0.693	This study
		Temperature (°C)		40.4			
		Inoculum size (%, v/v)		6.96			
		Shaking speed (rpm)		120			

*ND* not determined

## Conclusion

In this paper, we have presented our study on optimization of the medium and fermentation parameters for enhancing the enzyme production by *B. amyloliquefaciens* SS35. The statistical experimental design applied for medium optimization revealed that medium components providing carbon and nitrogen sources (viz. CMC, yeast extract, and peptone) are the most significant. The inorganic salts play relatively subordinate role. The statistical experimental design for optimization of fermentation parameters reveals the optimum range of these parameters. The optimization study of fermentation parameters reveals independent influence of the fermentation parameters, and overall, even the individual influence is only marginal, as compared to the effect of medium composition. The overall conclusion is that the enzyme production by *B. amyloliquefaciens* SS35 is mainly influenced by the medium components that supply carbon and nitrogen for the bacterial metabolism.

## Electronic supplementary material

Below is the link to the electronic supplementary material.

Supplementary Material.

The following supplementary material has been supplied with this manuscript: 1. Plackett–Burman experimental design for medium optimization; 2. Full factorial CCD matrix of 3 medium components; 3. Full factorial CCD matrix of 4 fermentation parameters; 4. Metabolic and energy requirements and stoichiometric coefficients to produce one mole of CMCase; 5. Amino acid sequence of cellulase (*eng*A) gene of *B. amyloliquefaciens*; 6. Pareto plot for Plackett–Burman analysis; 7. Desirability function plot showing the optimum levels of medium components, 8. Desirability function plot showing the optimum levels of fermentation parameters.

Supplementary material 1 (DOC 407 kb)

## Abbreviations

OVAT: One-variable-at-a-time
CCD: Central composite design
CMC: Carboxymethylcellulose
CMCase: Carboxymethylcellulase
