# Supplementary material for: Optimization of carboxymethylcellulase production from Bacillus amyloliquefaciens SS35
Source: 3 Biotech. 2013 Sep 6;4(4):411–24. doi: 10.1007/s13205-013-0169-6 (PMC4145630; doi:10.1007/s13205-013-0169-6)
Supplement: Supplementary file 1 — Supplementary material 1 (DOC 407 kb) [file 13205_2013_169_MOESM1_ESM.doc]

SUPPLEMENTARY MATERIAL WITH

Optimization of carboxymethylcellulase production from *Bacillus amyloliquefaciens* SS35

Shuchi Singh, Vijayanand S. Moholkar and Arun Goyal

Table S1. Plackett–Burman design in coded units and real values (in parenthesis) in g/L for six variables along with the CMCase activity

| Run order | CMC (*X*_1_) | Yeast extract (*X*_2_) | Peptone (*X*_3_) | K_2_HPO_4_ (*X*_4_) | MgSO_4_⋅7H_2_O  (*X*_5_) | NaCl (*X*_6_) | CMCase activity (U/mL) | |
| --- | --- | --- | --- | --- | --- | --- | --- | --- |
|  |  |  |  |  |  |  | Experimental | Predicted |
| 1 | −1 (10) | +1 (16) | +1 (10) | −1 (1) | −1 (0.1) | −1 (1) | 0.263 ± 0.030 | 0.281 |
| 2 | +1 (26) | −1 (8) | −1 (2) | −1 (1) | −1 (0.1) | +1 (4) | 0.366 ± 0.032 | 0.391 |
| 3 | −1 (10) | +1 (16) | +1 (10) | +1 (4) | +1 (0.4) | −1 (1) | 0.272 ± 0.014 | 0.274 |
| 4 | −1 (10) | −1 (8) | −1 (2) | +1 (4) | −1 (0.1) | +1 (4) | 0.155 ± 0.013 | 0.174 |
| 5 | −1 (10) | +1 (16) | −1 (2) | +1 (4) | −1 (0.1) | +1 (4) | 0.241 ± 0.012 | 0.258 |
| 6 | +1 (26) | +1 (16) | −1 (2) | −1 (1) | +1 (0.4) | +1 (4) | 0.478 ± 0.017 | 0.453 |
| 7 | +1 (26) | +1 (16) | −1 (2) | −1 (1) | −1 (0.1) | −1 (1) | 0.499 ± 0.027 | 0.460 |
| 8 | −1 (10) | +1 (16) | +1 (10) | −1 (1) | +1 (0.4) | +1 (4) | 0.267 ± 0.021 | 0.274 |
| 9 | +1 (26) | −1 (8) | −1 (2) | +1 (4) | +1 (0.4) | −1 (1) | 0.372 ± 0.023 | 0.369 |
| 10 | +1 (26) | −1 (8) | +1 (10) | +1 (4) | −1 (0.1) | −1 (1) | 0.431 ± 0.019 | 0.445 |
| 11 | −1 (10) | −1 (8) | −1 (2) | −1 (1) | +1 (0.4) | −1 (1) | 0.132 ± 0.018 | 0.121 |
| 12 | +1 (26) | −1 (8) | +1 (10) | −1 (1) | +1 (0.4) | +1 (4) | 0.413 ± 0.025 | 0.422 |
| 13 | +1 (26) | −1 (8) | +1 (10) | +1 (4) | +1 (0.4) | +1 (4) | 0.425 ± 0.023 | 0.438 |
| 14 | −1 (10) | +1 (16) | −1 (2) | +1 (4) | +1 (0.4) | +1 (4) | 0.223 ± 0.016 | 0.236 |
| 15 | +1 (26) | +1 (16) | −1 (2) | +1 (4) | +1 (0.4) | −1 (1) | 0.461 ± 0.023 | 0.453 |
| 16 | −1 (10) | −1 (8) | +1 (10) | −1 (1) | +1 (0.4) | −1 (1) | 0.173 ± 0.011 | 0.175 |
| 17 | −1 (10) | −1 (8) | −1 (2) | −1 (1) | −1 (0.1) | −1 (1) | 0.132 ± 0.012 | 0.143 |
| 18 | +1 (26) | +1 (16) | +1 (10) | −1 (1) | −1 (0.1) | +1 (4) | 0.528 ± 0.017 | 0.529 |
| 19 | −1 (10) | −1 (8) | +1 (10) | +1 (4) | −1 (0.1) | +1 (4) | 0.306 ± 0.022 | 0.227 |
| 20 | +1 (26) | +1 (16) | +1 (10) | +1 (4) | −1 (0.1) | −1 (1) | 0.516 ± 0.009 | 0.529 |
| ^*^Experimental values are mean ± SE (n = 3) | | | | | | | | |

Table S2. Full factorial central composite design matrix of three medium components in coded and actual (in parentheses) values and the response of CMCase activity

| Run order | CMC (*X*_1_) | Yeast extract (*X*_2_) | Peptone (*X*_3_) | CMCase activity (U/mL) | |
| --- | --- | --- | --- | --- | --- |
|  |  |  |  | Experimental | Predicted |
| 1 | 0 (18) | 0 (12) | 0 (6) | 0.456 ± 0.031 | 0.444 |
| 2 | +1 (22.75) | +1 (14.37) | +1 (8.37) | 0.500 ± 0.042 | 0.51 |
| 3 | 0 (18) | −α (8) | 0 (6) | 0.456 ± 0.031 | 0.444 |
| 4 | 0 (18) | 0 (12) | 0 (6) | 0.456 ± 0.031 | 0.444 |
| 5 | 0 (18) | 0 (12) | 0 (6) | 0.410 ± 0.073 | 0.419 |
| 6 | 0 (18) | 0 (12) | +α (10) | 0.425 ± 0.064 | 0.414 |
| 7 | −1 (13.24) | +1 (14.37) | −1 (3.62) | 0.430 ± 0.048 | 0.428 |
| 8 | 0 (18) | 0 (12) | 0 (6) | 0.411 ± 0.056 | 0.424 |
| 9 | 0 (18) | 0 (12) | 0 (6) | 0.289 ± 0.054 | 0.294 |
| 10 | +α (26) | 0 (12) | 0 (6) | 0.268 ± 0.036 | 0.26 |
| 11 | +1 (22.75) | −1 (9.62) | −1 (3.62) | 0.484 ± 0.062 | 0.461 |
| 12 | 0 (18) | 0 (12) | −α (2) | 0.148 ± 0.024 | 0.168 |
| 13 | −1 (13.24) | −1 (9.62) | −1 (3.62) | 0.530 ± 0.051 | 0.517 |
| 14 | −1(13.24) | +1 (14.37) | +1 (8.37) | 0.412 ± 0.046 | 0.395 |
| 15 | −α (10) | 0 (12) | 0 (6) | 0.431 ± 0.041 | 0.444 |
| 16 | −1 (13.24) | −1 (9.62) | +1 (8.37) | 0.457 ± 0.083 | 0.477 |
| 17 | +1 (22.75) | +1 (14.37) | −1 (3.62) | 0.431 ± 0.075 | 0.444 |
| 18 | 0 (18) | 0 (12) | 0 (6) | 0.279 ± 0.061 | 0.262 |
| 19 | 0 (18) | +α (16) | 0 (6) | 0.500 ± 0.086 | 0.513 |
| 20 | +1 (22.75) | −1 (9.62) | +1 (8.37) | 0.431 ± 0.078 | 0.444 |
| ^*^Experimental values are mean ± SE (n = 3) | | | | | |

Table S3. Full factorial central composite design matrix of 4 fermentation parameters in coded and actual (in parentheses) values and the response of CMCase activity

| Run order | Temperature (^o^C) (*X*_1_) | Medium pH (*X*_2_) | Shaking speed (rpm) (*X*_3_) | Inoculum size (%, v/v) (*X*_4_) | CMCase activity (U/mL) | |
| --- | --- | --- | --- | --- | --- | --- |
|  |  |  |  |  | Experimental | Predicted |
| 1 | 0 (37.5) | −α (5) | 0 (185) | 0 (5) | 0.643 ± 0.041 | 0.651 |
| 2 | 0 (37.5) | 0 (7.5) | 0 (185) | 0 (5) | 0.642 ± 0.035 | 0.638 |
| 3 | +1 (43.75) | +1 (8.75) | +1 (217.5) | −1 (3.5) | 0.581± 0.056 | 0.575 |
| 4 | 0 (37.5) | 0 (7.5) | 0 (185) | 0 (5) | 0.637 ± 0.062 | 0.638 |
| 5 | −1 (31.25) | +1 (8.75) | +1 (217.5) | −1 (3.5) | 0.576 ± 0.047 | 0.578 |
| 6 | 0 (37.5) | 0 (7.5) | 0 (185) | −α (2) | 0.612 ± 0.047 | 0.617 |
| 7 | +1 (43.75) | +1 (8.75) | −1 (152.5) | −1 (3.5) | 0.603 ± 0.029 | 0.600 |
| 8 | 0 (37.5) | 0 (7.5) | −α (120) | 0 (5) | 0.678 ± 0.031 | 0.685 |
| 9 | −α (25) | 0(7.5) | 0 (185) | 0 (5) | 0.514 ± 0.037 | 0.507 |
| 10 | 0 (37.5) | 0 (7.5) | 0 (185) | 0 (5) | 0.627 ± 0.045 | 0.638 |
| 11 | +1 (43.75) | −1 (6.25) | −1 (152.5) | +1 (6.5) | 0.673 ± 0.052 | 0.659 |
| 12 | 0 (37.5) | 0 (7.5) | +α (250) | 0 (5) | 0.658 ± 0.082 | 0.661 |
| 13 | 0 (37.5) | 0 (7.5) | 0 (185) | 0 (5) | 0.634 ± 0.059 | 0.638 |
| 14 | −1 (31.25) | −1 (6.25) | +1 (217.5) | +1 (6.5) | 0.632 ± 0.081 | 0.623 |
| 15 | +1 (43.75) | +1 (8.75) | +1 (217.5) | +1 (6.5) | 0.604 ± 0.091 | 0.597 |
| 16 | 0 (37.5) | 0 (7.5) | 0 (185) | 0 (5) | 0.638 ± 0.038 | 0.638 |
| 17 | +α (50) | 0 (7.5) | 0(185) | 0 (5) | 0.523 ± 0.082 | 0.54 |
| 18 | −1 (31.25) | −1 (6.25) | +1 (217.5) | −1 (3.5) | 0.621 ± 0.081 | 0.626 |
| 19 | 0 (37.5) | 0 (7.5) | 0 (185) | +α (8) | 0.632 ± 0.067 | 0.637 |
| 20 | +1 (43.75) | −1 (6.25) | −1 (152.5) | −1 (3.5) | 0.642 ± 0.083 | 0.638 |
| 21 | +1 (43.75) | −1 (6.25) | +1 (217.5) | −1 (3.5) | 0.617 ± 0.079 | 0.608 |
| 22 | −1 (31.25) | +1 (8.75) | −1 (152.5) | +1 (6.5) | 0.575 ± 0.060 | 0.572 |
| 23 | −1 (31.25) | +1 (8.75) | −1 (152.5) | −1 (3.5) | 0.573 ± 0.058 | 0.573 |
| 24 | −1 (31.25) | −1 (6.25) | −1 (152.5) | −1 (3.5) | 0.631 ± 0.092 | 0.626 |
| 25 | −1 (31.25) | +1 (8.75) | +1 (217.5) | +1 (6.5) | 0.571 ± 0.067 | 0.577 |
| 26 | +1 (43.75) | +1 (8.75) | −1 (152.5) | +1 (6.5) | 0.625 ± 0.092 | 0.622 |
| 27 | −1 (31.25) | −1 (6.25) | −1 (152.5) | +1 (6.5) | 0.615 ± 0.054 | 0.623 |
| 28 | +1 (43.75) | −1 (6.25) | +1 (217.5) | +1 (6.5) | 0.628 ± 0.072 | 0.630 |
| 29 | 0 (37.5) | 0 (7.5) | 0 (185) | 0 (5) | 0.643 ± 0.081 | 0.638 |
| 30 | 0 (37.5) | 0 (7.5) | 0 (185) | 0 (5) | 0.643 ± 0.072 | 0.638 |
| 31 | 0 (37.5) | +α (10) | 0 (185) | 0 (5) | 0.564 ± 0.052 | 0.566 |
| ^*^Experimental values are mean ± SE (n = 3) | | | | | | |

Table S4. Metabolic and energy requirements and stoichiometric coefficients to produce one mole of CMCase (Endoglucanase) by *Bacillus amyloliquefaciens.* The amino acid composition of cellulase was obtained from European Nucleotide Archive (Sequence: AF363635.1). G3P Glycerol-3-phosphate, R5P Ribose-5-phosphate, E4P erythrose-4-phosphate, PEP phosphoenol pyruvate, PYR pyruvate, ACCoA acetyl coenzyme A, AKG *α*-ketoglutarate, OAA oxaloacetate.

| **Amino acid** | **Moles** | **G3P** | **R5P** | **E4P** | **PEP** | **PYR** | **ACCOA** | **AKG** | **OA** | **NH3** | **ATP** | **NADH** | **NADPH** | **CO_2_** |
| --- | --- | --- | --- | --- | --- | --- | --- | --- | --- | --- | --- | --- | --- | --- |
| Alanine | 35 | -- | -- | -- | -- | 35 | -- | -- | -- | 35 | -- | -- | 35 | -- |
| Arginine | 15 | -- | -- | -- | -- | -- | -- | 15 | -- | 60 | 105 | -15 | 60 | -- |
| Asparagine | 38 | -- | -- | -- | -- | -- | -- | -- | 38 | 76 | 114 | -- | 38 | -- |
| Asparate | 32 | -- | -- | -- | -- | -- | -- | -- | 32 | 32 | -- | -- | 32 | -- |
| Cysteine | 3 | -- | -- | -- | -- | -- | -- | -- | -- | -- | -- | -- | -- | -- |
| Glutamine | 22 | -- | -- | -- | -- | -- | -- | 22 | -- | 44 | 22 | -- | 22 | -- |
| Glutamate | 19 | -- | -- | -- | -- | -- | -- | 19 | -- | 19 | -- | -- | 19 | -- |
| Glycine | 46 | 46 | -- | -- | -- | -- | -- | -- | -- | 46 | -- | -46 | 46 | -46 |
| Histidine | 10 | -- | 6 | -- | -- | -- | -- | -- | -- | 30 | 60 | -30 | 10 | 10 |
| Isoleucine | 32 | -- | -- | -- | -- | 32 | -- | -- | 32 | 32 | 64 | -- | 160 | -32 |
| Leucine | 36 | -- | -- | -- | -- | 72 | 36 | -- | -- | 36 | -- | -36 | 72 | -- |
| Lysine | 43 | -- | -- | -- | -- | 43 | -- | -- | 43 | 86 | 86 | -- | 172 | -- |
| Methionine | 9 | -- | -- | -- | -- | -- | -- | -- | 9 | 9 | 63 | -- | 72 | 9 |
| Phenylalanine | 13 | -- | 13 | -- | 26 | -- | -- | -- | -- | 13 | 13 | -- | 26 | -- |
| Proline | 21 | -- | -- | -- | -- | -- | -- | 21 | -- | 21 | 21 | -- | 63 | -- |
| Serine | 34 | 34 | -- | -- | -- | -- | -- | -- | -- | 34 | -- | -34 | 34 | -- |
| Threonine | 34 | -- | -- | -- | -- | -- | -- | -- | 34 | 34 | 68 | -- | 102 | -- |
| Tryptophane | 13 | -- | 13 | 13 | 13 | -- | -- | -- | -- | 26 | 65 | -26 | 39 | -- |
| Tyrosine | 18 | -- | -- | 18 | 36 | -- | -- | -- | -- | 18 | 18 | -18 | 36 | -- |
| Valine | 26 | -- | -- | -- | -- | 52 | -- | -- | -- | 26 | -- | -- | 52 | -- |
| Peptide bonds | 498 | -- | -- | -- | -- | 234 | 36 | 77 | 188 | -- | 1096 | -- | -- | -- |
| **Stoichiometric coefficient** | 1 | 80 | 32 | 31 | 75 | 139 | 15 | 32 | 72 | 677 | 1795 | -205 | 1055 | -59 |

Adopted in modified form from: P. Unrean, N. H. A. Nguyen, Metabolic pathway analysis and kinetic studies for production of nattokinase in *Bacillus subtilis*, Bioprocess Biosyst Eng (2013) 36:45–56.

**Amino acid sequence of cellulase (*eng*A) gene of *B. amyloliquefaciens***

MKRSISIFITCLLIAVLTMGGLLPSPASAAGTKTPVAKNGQLSIKDTQLVNRDGKAVQLKGISSHGLQWYGDFVNKDSLKWLRDDWGITVFRAAMYTADGGYIDNPSVKNKVKEAVEAAKELGIYVIIDWHILNDGNPNQNKEKAKEFFKEMSSLYGNTPNVIYEIANEPNGDVNWKRDIKPYAEEVISVIRKNDPDNIIIVGTGTWSQDVNDAADDQLKDANVMYALHFYAGTHGQSLRDKANYALSKGAPIFVTEWGTSDASGNGGVFLDQSREWLNYLDSKNISWVNWNLSDKQESSSALKPGASKTGGWPLTDLTASGTFARENIRGTKGSTKDGPETPAQDNPTQEKGVSVQYKAGYGRVNSNQIRPQLHMKNNGNTKVDLKGVTARYWYNTKNKGQNFDCDYTQIGCGNLTHKFVTLHKPKQDADTYLELGFKTGTLSPGASTGNIQLRLPMMTGAIMHKATIIPFSNQIHLKQREKSHYISQGKLIWGTEPN

Reference: European Nucleotide Archive, Sequence: AF363635.1

Organism: *Bacillus amyloliquefaciens*, Strain: UMAS1002

(available on webpage: http://www.ebi.ac.uk/ena/data/view/AAL99668)


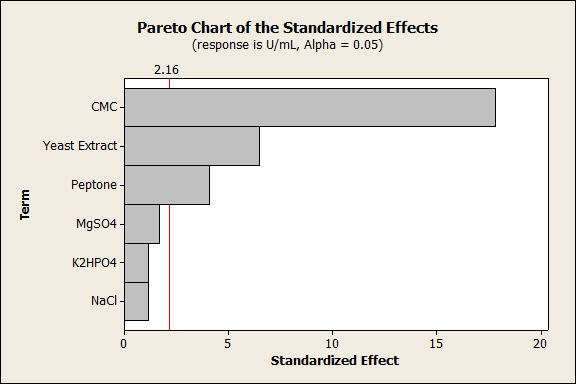


Figure S1. Pareto plot for Plackett–Burman analysis


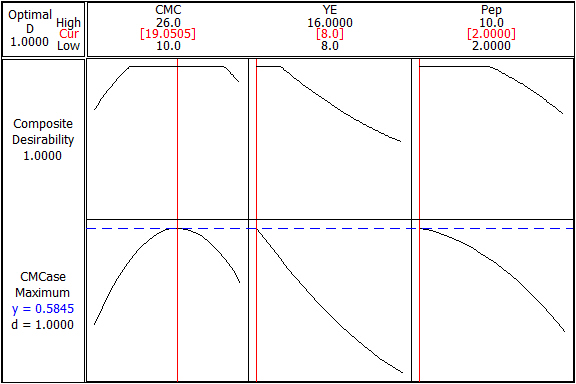


Figure S2. Desirability function plot showing the optimum levels of medium components


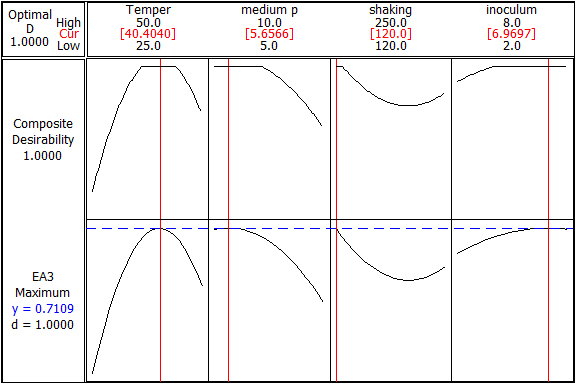


Figure S3. Desirability function plot showing the optimum levels of fermentation parameters
